# Broadening the Spectrum of Adulthood X-Linked Adrenoleukodystrophy: A Report of Two Atypical Cases

**DOI:** 10.3389/fneur.2019.00070

**Published:** 2019-02-06

**Authors:** Matteo Foschi, Veria Vacchiano, Patrizia Avoni, Alex Incensi, Stella Battaglia, Vincenzo Donadio, Elena Panzeri, Maria Teresa Bassi, Rocco Liguori, Giovanni Rizzo

**Affiliations:** ^1^Department of Biomedical and Neuromotor Sciences, University of Bologna, Bologna, Italy; ^2^UOC Clinica Neurologica, IRCCS Istituto delle Scienze Neurologiche di Bologna, Bologna, Italy; ^3^UOC Neuroradiologia, IRCCS Istituto delle Scienze Neurologiche di Bologna, Bologna, Italy; ^4^Laboratory of Molecular Biology, Scientific Institute IRCCS E. Medea, Lecco, Italy

**Keywords:** adrenoleukodystrophy, small fiber neuropathy, skin biopsy, MRI, adrenomyelopathy, brain development defects, *ABCD1*, very long chain fatty acids

## Abstract

X-linked adrenoleukodystrophy (x-ALD) is a rare genetic disorder caused by a mutation in the *ABCD1* gene, which encodes for a peroxisomal very long chain fatty acid transporter. Clinically, x-ALD can present a wide spectrum of different phenotypes: asymptomatic carriers, Addison only, cerebral x-ALD, and myelopathy with/without evidence of peripheral axonopathy (Adrenomyeloneuropathy). We report on two cases of adult x-ALD, with atypical phenotypes: **(Case 1)** A 37-years-old male with a 2-years-long history of spastic paraparesis, urinary urgency, and subclinical adrenocortical insufficiency. As an atypical finding, the MRI showed multiple congenital brain development defects. **(Case 2)** A 63-years-old male with a previous diagnosis of Addison disease, with a 6-years-long history of spastic paraparesis. Two years later, he complained of severe and disabling burning pain in his feet. A nerve conduction study was normal, but a skin biopsy revealed autonomic and somatic small fiber neuropathy. In both cases, genetic testing disclosed hemizygous mutation in *ABCD1* associated with x-ALD: c.1394-2A > G and p.(Thr254Met), respectively. While case 1 supports the key role of peroxisome functions in brain development, case 2 points to a possible selective and clinically relevant peripheral small fiber degeneration in x-ALD myelopathy.

## Introduction

X-linked adrenoleukodystrophy (x-ALD) is a rare genetic disorder with a wide spectrum of different clinical phenotypes (1). The disease is caused by the impairment of peroxisomal beta-oxidation of very long chain fatty acids (VLCFAs), which accumulate in the central nervous system (CNS), adrenal cortex and testes (2–4). The metabolic abnormality is caused by mutations in the *ABCD1* gene, which is located in the X chromosome. *ABCD1* encodes for a peroxisomal membrane transporter of (adrenoleukodystrophy protein, ALDP) (5–7).

Clinically, x-ALD phenotypes include asymptomatic carriers, Addison only, childhood, adolescent, and adult cerebral x-ALD, myelopathy with or without evidence of peripheral axonal neuropathy (Adrenomyeloneuropathy, AMN) (1, 8). The pathophysiological hallmark of the neurological involvement in x-ALD, consists of a combination of slowly progressive spinal cord and peripheral nerve damage, with or without CNS inflammatory demyelination (9, 10). The latter is typical of cerebral x-ALD and it seems to have a different pathogenesis from the chronic myelopathy and peripheral axonopathy of adult x-ALD (1, 10, 11).

This paper reports on two patients with adult x-ALD showing unusual clinical features: (1) congenital brain development defects and (2) clinically relevant x-ALD related small fiber neuropathy (SFN).

Both patients explicitly agreed to their inclusion in this retrospective observational case report and gave written informed consent for publication.

## Case Reports

### Case 1

**Figure 1:**
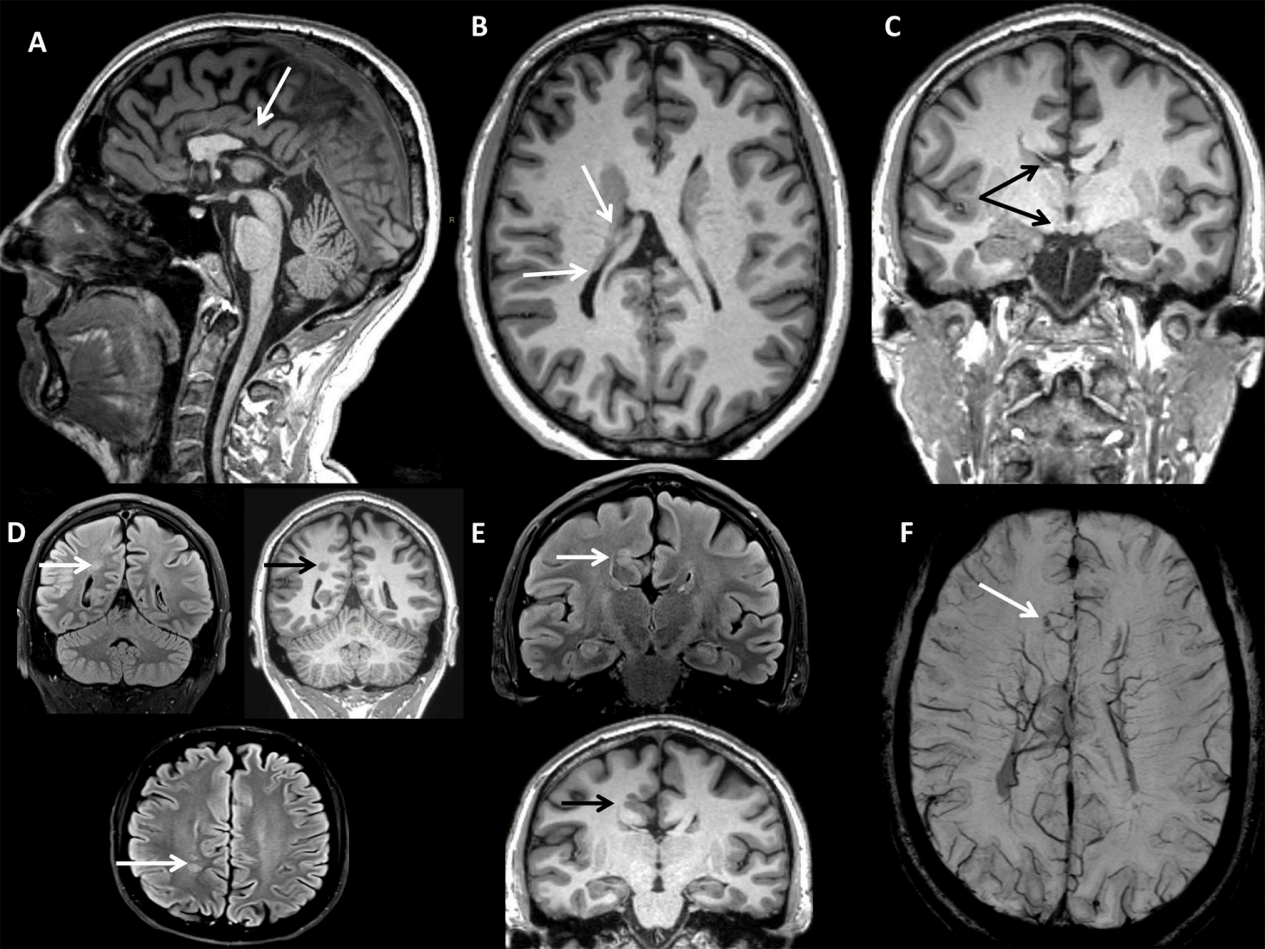
BRAIN MRI (CASE 1): **(A)** Sagittal 3D Magnetization Prepared Rapid Acquisition Gradient Echo (MPRAGE) T1-w with axial **(B)**, and coronal **(C)** reconstructions showing partial corpus callosum agenesis, right fornix, and mammillary body hypoplasia, hypogenesis of bilateral lateral ventricles (colpocephaly). **(D)** Turbo Spin Echo Fluid-Attenuated Inversion Recovery (TSE FLAIR) T2-w axial and coronal (FLAIR T2-w & T1-w) scans showing nodular heterotopic foci. **(E)** TSE FLAIR T2-w & T1-w coronal scan showing right frontal parasagittal cortical thickening. **(F)** Susceptibility Weighted Imaging (SWI) axial scan with Maximum Intensity Projection (MIP) reconstruction showing right frontal parasagittal venous drainage abnormality with telangiectasia.

A 37-years-old male caught our attention due to the onset of progressive gait difficulties caused by a rigidity and weakness affecting both legs from the age of 35. At the time, he complained of urinary urgency with incontinence and erectile dysfunction. His family history was negative for neurological or endocrinological diseases. He had normal psychomotor development without learning disabilities and did not report cognitive symptoms. The neurological examination (NE) showed mild dysarthria, spastic paraparesis with a wide-based spastic gait. Deep tendon reflexes were diffusely brisk with a bilateral Achilles clonus and Babinski sign. A brain magnetic resonance imaging (MRI) scan showed multiple congenital brain development defects (Figure 1): posterior commissure agenesis, right fornix, and ipsilateral mammillary body hypoplasia, colpocephaly, right frontal parasagittal cortical thickening, two periventricular nodular heterotopic foci in the right parietal areas, and two venous drainage abnormalities in the left cerebellar hemisphere and right frontal lobe, respectively. A neuropsychological evaluation revealed no abnormalities. Electroencephalography (EEG) did not show any epileptiform discharges. A spinal MRI showed spinal cord atrophy. Electromyography (EMG) did not reveal any abnormal finding. Somatosensory evoked potentials (SEPs) showed increased central conduction time from the right arm and the left leg. No response was recorded from the right leg. Motor evoked potentials (MEPs) were absent in both legs. Visual evoked potentials (VEPs), and optic coherence tomography (OCT) were unremarkable. We used a multi-gene panel for hereditary spastic paraplegia and other motor neuron diseases (Supplementary Material). Genetic analysis revealed the presence of the hemizygous mutation c.1394-2A > G in the *ABCD1* gene, leading to the diagnosis of x-ALD. Hematochemical examination disclosed normal cortisol levels with an increased adrenocorticotrophic hormone (ACTH; 352 pg/mL, n.v. 5–60 pg/mL), consistent with subclinical adrenocortical insufficiency. VLCFA plasma levels were increased. A multi-gene panel testing for cortical development defects excluded other possible genetic causes (Supplementary Material).

### Case 2

**Figure 2:**
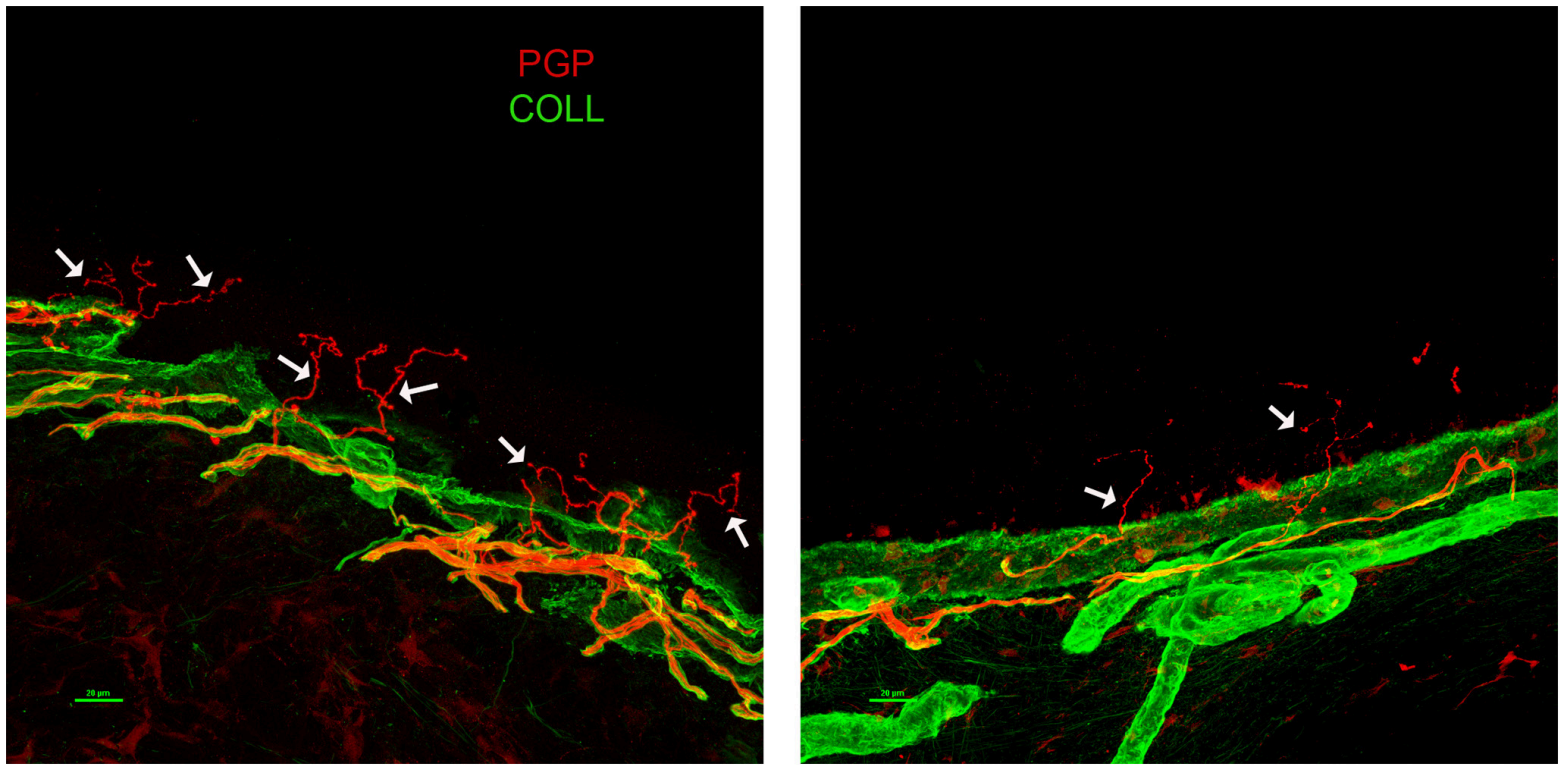
Skin Biopsy Immunoflorescence (Case 2) showing prevalently somatic small fiber neuropathy (SFN). A confocal study of epidermal innervation in the patient and a control subject: leg epidermal innervation disclosed by confocal microscope (×40) in an age-matched control subject **(left panel)**, and the x-ALD patient case 2 **(right panel)**. Nerve fibers are marked in red by a pan-neuronal marker PGP 9.5 whereas, the collagen staining is shown in green. The basement membrane separating epidermis from dermis is marked by collagen staining. Free-ending PGP immunoreactive fibers are evident in the epidermis of the control (arrows) whereas, the patient showed a marked decrease of such fibers, often fragmented (arrows). PGP, protein gene product; COLL, collagen.

A 63-years-old male with a 6-years-long history of progressive gait impairment, received a diagnosis of Addison's disease at the age of 13, and had been chronically treated with cortone acetate from the age of 53. From the age of 57, he noticed a progressive tendency to drag both his feet, which is associated with orthostatic imbalance. His family history was negative for neurological or endocrinological diseases. Psychomotor development was normal and the patient did not report cognitive symptoms. On admission, NE showed diffuse skin pigmentation, and spastic paraparesis (right > left). The deep tendon reflexes were brisk with bilateral Achilles clonus, Babinski, and a right-hand Hoffman sign. Hematochemical investigations, including liver and renal functions, vitamin B12, folic acid, creatinine phosphokinase (CPK), thyroid hormone levels, and a complete screening for autoimmune disease, were all unremarkable. EMG excluded a peripheral neuropathy. SEP showed an increased latency in the central responses from the upper and lower limbs. No motor responses were recorded from the lower limbs. A brain MRI showed T2-hyperintensity of the corticospinal tracts (left > right) with a bilateral hypointensity of the pre-central gyrus in susceptibility weighted imaging (SWI) sequences. A spinal MRI showed atrophy of the spinal cord. Neuropsychological evaluation uncovered no abnormalities. Plasma levels of VLCFA were increased. Genetic analysis of the *ABCD1* gene, disclosed the presence of the hemizygous base change c.761C > T, leading to the amino acid substitution p.(Thr254Met). This change is known in ClinVar, as likely pathogenic and classified pathogenic, according to the ACMG guidelines for variant classification, confirming the diagnosis of x-ALD. From the age of 65, the patient started to complain of a severe burning pain and painful dysesthesia affecting the lower limbs and feet. Within a few months, the pain rapidly became his main complaint impairing his quality of life. The patient was treated with common analgesics, gabapentin, amitriptyline, duloxetine, and cannabis without substantial improvement or side effects. High doses of pregabalin mildly attenuated the symptoms. EMG was repeated with negative results. Therefore, the patient underwent a skin biopsy. The immunofluorescence (IF) analysis (12) revealed a prevalently somatic SFN (Figure 2). We repeated blood tests including hepatic and renal function, thyroid hormones levels, serological screening for infectious diseases, and a glucose challenge test. All tests turned out to be in range, excluding the presence of risk factors potentially associated with SFN (13). His family history was negative for symptoms possibly related with SNF. We performed whole exome sequencing to search for the possible presence of concomitant mutations/variants in other genes that could explain the complex clinical phenotype. Among genes causing hereditary neuropathies, whole exome analysis identified only two heterozygous variants in *SBF1* (c.3044G>A, p.Arg1015Gln-rs372268920) and *WNK1* (c.2228C > T, p.Pro743Leu -rs528772088), genes with a very low allele frequency in the ExAC database (0.0003 and 0.0008, respectively). In both cases bioinformatics analysis predict likely deleterious effects on protein function. None of them are known in the ClinVar database. However, both genes are associated with recessive diseases: *SBF1* with Charcot Marie Tooth disease type 4B3, (14) and *WNK1* with a hereditary sensory and autonomic neuropathy, type 2 (15). Therefore, the sole presence of these variants may somewhat contribute to the SFN phenotype, but cannot be considered pathogenic mutations. Furthermore, given the known relationship between *WNK1* mutations and pseudohypoaldosteronism type 2 (16), the endocrinological history was deepened, by searching for specific features (hypertension, hyperkalemia, or hyperchloremic metabolic acidosis). The patient had no antecedents of suggestive symptoms and repeated blood, and urine analyses never showed electrolytes or pH alterations.

The clinical and demographic characteristics of case 1 and 2 are summarized in Table 1, along with the results of the principal diagnostic investigations.

**Table 1:** Clinical features and diagnostic investigations of case 1 and case 2.

	**Sex**	**Age**	**Clinical phenotype**	**Endocrine involvement**	**Neurological symptoms**	Diagnostic investigations^*^				
						**HCI**	Brain MRI^*^	**EMG**	**Skin biopsy**	**ABCD1 mutation**
Case 1	M	37	Myelopathy	Subclinical hypocortisolism	35 years ▸ urinary urgency and spastic paraparesis	**↑**ACTH ↔ Cortisol **↑**VLCFA	Posterior commissure agenesis, right fornix and mammillary body hypoplasia, colpocephaly, cortical dysplasia, periventricular heterotropia^$^	Normal	NA	c.1394-2A>G
Case 2	M	63	Myelopathy	Addison (from childhood)	57 years▸ spastic paraparesis 65 years ▸ lower extremities burning pain and dysesthesia	**↑**ACTH **↓**Cortisol **↑**VLCFA	T2 corticospinal tracts hyperintensity, bilateral hypointensity of the pre- central gyrus (SWI)	Normal	SFN (prevalently somatic)	p.(Thr254Met)

*HCI, haematochemical investigations; ACTH, adrenocorticotropic hormone; VLCFA, very long chain fatty acids; NA, not available; SWI, susceptibility weighted image; SFN, small fiber neuropathy*.

* *An in-toto spine MRI was performed in both cases showing spinal cord atrophy*.

$ *Electroencephalography (EEG) excluded epileptiform activity*.

## Discussion and Conclusions

We have described two cases of adult x-ALD and their atypical clinical features. Case 1 clinically corresponds to a pure adrenomyelopathy without cerebral demyelination. The patient carried the c.1394-2A > G mutation of the *ABCD1* gene. This mutation has previously been reported only in one Indian patient affected by childhood cerebral x-ALD and without evidence of neuronal migrations defects (17).

Although not experimentally proven, at the transcript level, the base change alters the wild type (WT) acceptor site, most probably affecting *ABCD1* RNA splicing. With these limits, our cases present two peculiarities: (1) the presence of brain development defects on the MRI, not previously observed in the adult x-ALD spectrum; and (2) the late onset with a typical adrenomyelopathy phenotype. Brain development alterations can be related to specific metabolic dysfunctions. In particular, they may result from a complete loss of all peroxisomal functions, or from defects in individual enzymatic reactions (18). Indeed, pathological aberrations of the central nervous system are prominent features in most peroxisomal disorders. Specifically, peroxisome biogenesis disorders (PBD) include: (1) Zellweger spectrum disorders *(Zellweger syndrome, neonatal adrenoleukodystrophy, and infantile Refsum)*, related to mutations in 13 different peroxin (PEX) genes *(PEX1, PEX2, PEX3, PEX5, PEX6, PEX10, PEX11b, PEX12, PEX13, PEX14, PEX16, PEX19, PEX26)* and (2) rhizomelic chondrodysplasia punctata (RCDP) type 1, caused by mutations in the *PEX 7* gene (18). In Zellweger syndrome, typical brain pathological features are abnormalities in the cytoarchitecture of the cerebral cortex with microgyric and pachygyric areas. The most frequent finding is a local thickening of small convolutions around the central sulcus (centrosylvian pachygyria), sometimes with an excess of local convolutions in the same area (polymicrogyria) (19). The heterotopic localization of Purkinje cells within the cerebellar white matter is often observed (18). Several metabolic diseases related to a single enzyme deficiency (SED) can cause neuronal migration defects (20, 21). For instance, neocortical dysplasia has been reported in some cases of acyl-CoA oxidase deficiency, in association with typical cerebral and/or cerebellar white matter abnormalities on the MRI (20). Another example is peroxisomal D-bifunctional protein (DBP) deficiency (21). These two conditions share a disruption in the peroxisomal VLCFA degradation pathway, with x-ALD. Less severe cerebral migratory abnormalities have occasionally been reported in neonatal x-ALD (22). These observations suggest that multiple peroxisomal functions are required for proper brain development.

The congruence of the biochemical patterns of VLCFAs in x-ALD, Zellweger spectrum disorder and SED syndromes (19–21) suggests that their accumulation could cause specific morphogenesis abnormalities. Pathologically increased VLCFAs have been related to direct and indirect biochemical disruptions (22). Specifically, VLCFAs inhibit intracellular biochemical processes and interfere with the metabolism of other unsaturated fatty acids (such as arachidonic acid–C20:4) that play an important role in histogenesis (22). We can only speculate that the neurogenesis aberrations rarely reported in x-ALD, can be explained by the same mechanisms.

Case 2 presented adrenocortical deficiency, myelopathy, and a painful syndrome due to SFN. SFN has recently been observed as a frequent finding in x-ALD, although only in a small case series (23). It appears to be more prevalent in older subjects with an AMN phenotype (23). Similar to large fiber degeneration in AMN, SFN probably starts in an early asymptomatic stage and progresses with age. The pathogenesis of x-ALD-related SFN is still unclear. The frequent association with peripheral axonopathy suggests a myelopathy-related dying back process (23) but small fibers are rarely affected selectively, as in our case. Our report represents the first histologic characterization of x-ALD related SFN through a skin biopsy analysis with IF (12). The negativity of all tests performed to exclude other possible secondary causes (diabetes, infectious diseases, hepatic, or thyroid alterations, autoimmunity) (13) support a probable relationship with x-ALD. The whole exome sequencing excluded concomitant mutations in other genes causing hereditary neuropathies, except for two heterozygous variants in the *SBF1* and *WNK1* genes, which are both involved in different forms of recessive neuropathies (CMT4B3 and HSN2, respectively) (14, 15). Although not causative, we cannot exclude the possibility that these variants contribute to the phenotypic component of SFN observed in our patient. Indeed, carriers of *WNK1* recessive mutations show greater sensitivity to thermal and cold pain stimuli (24). However, although *WNK1* mutations are associated with autosomal dominant pseudohypoaldosteronism (16), our patient had no clinical or laboratoristic features of pseudohypoaldosteronism. Unfortunately, we did not have access to parental DNA to assess the segregation of all these variants.

Our skin specimen revealed a prevalently somatic small fiber loss. The somatic subtype accounts for about 30% of all peripheral small fibers (25). In contrast to autonomic fibers, somatic fibers are thicker and sparsely myelinated (25, 26). Therefore, the most plausible hypothesis is that somatic fibers undergo the same degenerative process, affecting the large myelinated nerves in the AMN, though this occurs at an earlier stage of disease progression. Another peculiarity of our report is the clinical presentation with predominant SFN-related symptoms (pain and dysesthesia unresponsive to commonly used therapies), in contrast to previously reported samples (23) in which SFN was asymptomatic or paucisymptomatic. The patient did not report any signs or symptoms that might be linked to dysautonomia (such as gastrointestinal motility disturbances, orthostatic hypotension, pupillary abnormalities, or sweating alterations). Further studies, including routinely performed skin biopsy analyses, are needed to clarify the actual prevalence of SFN in adult x-ALD with/without AMN. These data (along with histopathology) should contribute to a more complete characterization of x-ALD-related SFN, to improve the understanding of the pathogenic mechanisms underlying small fiber involvement.

In conclusion, while noting that extensive genetic analyses should be interpreted with caution in patients whose symptoms are related to different coexisting causes, our two cases expand the observed clinical heterogeneity of adult x-ALD, including the possible presence of brain development defects and clinically relevant SFN.

## Author Contributions

MF, VV, and GR collected clinical data and drafted the manuscript. RL and PA contributed to the clinical data collection and clinical management of the patients, and to the manuscript revision. MB and EP performed and interpreted genetic analyses, and revised the manuscript. VD and AI performed skin biopsy analysis, created Figure 2, and contributed to the revision. SB performed MRI, created Figure 1, and revised the manuscript. All authors approved the final version of the manuscript and agree to be accountable for all aspects of the work in ensuring that questions related to the accuracy or integrity of any part of the work are appropriately investigated and resolved.

### Conflict of Interest Statement

The authors declare that the research was conducted in the absence of any commercial or financial relationships that could be construed as a potential conflict of interest.
