# Supplementary material for: Broadening the Spectrum of Adulthood X-Linked Adrenoleukodystrophy: A Report of Two Atypical Cases
Source: Front Neurol. 2019 Feb 6;10:70. doi: 10.3389/fneur.2019.00070 (PMC6372518; doi:10.3389/fneur.2019.00070)
Supplement: Supplementary file 1 [file Table_1.docx]

**Supplementary material**

**Methods: genetic analyses**

**Case 1** After obtaining informed consent from the proband, genomic DNA was prepared from blood using a standard method. The proband’s DNA was screened using a targeted next generation sequencing approach, with a customized panel including 185 genes: all known causative genes for HSP, the known genes for recessive ataxia and for spinocerebellar ataxias, the most frequently mutated genes in neuropathies and the known genes for familial ALS (the genes’ list is reported below). Patient 1 was also screened with a panel specific for malformations of the cortical development (genes’ list is reported below). The targeted regions were designed to include coding exons with intronic 50 bp flanking sites and 3′ and 5′ untranslated regions (UTRs) using the SureDesign system (Agilent Technologies, Santa Clara, CA, USA). The sequencing libraries were prepared from genomic DNA, by using a Sure Select enrichment system (Agilent Technologies). Targeted libraries were run on the MiSeq platform according to the manufacturer's instructions (Illumina, San Diego, CA, USA).

ANNOVAR was used for annotation against the RefSeq database and the Single Nucleotide Polymorphism databases. The filtering strategy we applied led us to select only variants located in the coding regions, including the splice site (synonymous variants were excluded), variants that exhibited a minor allele frequency of less than 1% or that were not present in variant databases, including those of the 1000 Genomes Project, the Exome Aggregation Consortium (ExAC) and the NHLBI exome sequencing project ESP6500. On average, 98,66% and 99,3 % of bases were covered by at least 10 and 20 sequence reads, respectively. The mean read depth of the targeted regions was 824,34X. We used Polyphen2 and SIFT and Human Splicing Finder v.3.1 to assess the functional effects of the variants. After filtering, we performed Sanger sequencing to confirm the variants detected through a targeted sequencing analysis.

Genes included in multi-gene testing for hereditary spastic paraplegia and other motor neuron diseases:

AARS, ABCD1, ADD3, AFG3L2, ALDH18A1, ALS2, AMPD2, ANO10, AP4B1, AP4E1, AP4M1, AP4S1, AP5Z1, APTX, ARHGEF10, ARL6IP1, ARSI, ATL1, ATL3, ATM, ATN1, ATP13A2, ATXN10, B4GALNT1, BICD2, BSCL2, C10ORF2, C12ORF65, C19ORF12, CABC1, CACNA1A, CACNB4, CAPN1, CASK, CCDC88C, CHMP1A, COASY, CP, CPT1C, CYP27A1, CYP2U1, CYP7B1, DARS, DARS2, DCAF17, DCTN1, DDHD1, DDHD2, DGAT2, DNM2, DNMT1, DYNC1H1, EEF2, EGR2, ELOVL4, ELOVL5, ENTPD1, ERLIN1, ERLIN2, EXOSC3, FA2H, FGD4, FGF14, FIG4, FLRT1, FMR1, FTL, FXN, GARS, GBA2, GDAP1, GJB1, GJC2, GRID2, GSN, HEXA, HEXB, HSPB1, HSPB3, HSPB8, HSPD1, IBA57, IFRD1, IGHMBP2, ITPR1, KANK1, KCNA1, KCNC3, KCND3, KIAA0196, KIAA0226, KIF1A, KIF1B, KIF1C, KIF5A, L1CAM, LITAF, LMNA, MAG, MARS, MARS2, MFN2, MME, MORC2, MPZ, MRE11A, MTPAP, MTTP, NEFL, NEK1, NIPA1, NOP56, NPC1, NT5C2, OPA1, OPTN, PANK2, PDYN, PEX10, PEX7, PGAP1, PHF21A, PHYH, PLA2G6, PLP1, PMM2, PMP22, PNPLA6, POLG, PRKCG, PRRT2, RAB3GAP2, RAB7A, RARS2, REEP1, REEP2, RNF170, RTN2, SACS, SCP2, SEPSECS, SETX, SH3TC2, SIGMAR1, SIL1, SLC1A3, SLC33A1, SOD1, SPAST, SPG11, SPG20, SPG21, SPG7, SPTBN2, STUB1, SYNE1, SYNE2, TBK1, TBP, TDP1, TECPR2, TFG, TGM6, TK2, TMEM240, TRIM2, TRPC3, TRPV4, TSEN2, TSEN34, TSEN54, TTBK2, TTPA, UBR4, USP8, VAMP1, VAPB, VCP, VPS37A, VRK1, WDR48, YARS, ZFR, ZFYVE26, ZFYVE27.

Genes included in multi-gene testing for cortical development malformations:

AKT3, ALX1, ALX3, ALX4, AMPD2, ARFGEF2, ARID1B, ARX, ASPM, ATR, ATRX, B3GALTL, BRPF1, c12orf57, C6orf70, CASK, CCND2, CDK5R, AP2, CDON, CENPJ, CEP170, CHMP1A, COL4A1, CREBBP, CYP11A1, DCHS1, SCLK1, DCX, DHCR24, DHCR7, DIS3L2, DISC1, DISP1, DLL1, DMRTA2, DYNC1H1, DYRK1A, EARS2, EFNB1, EMX1, EOMES, EP300, ERBB4, ERMARD, EXOSC3, FAM36A, FGF8, FGFR1, FGFR2, FLNA, FOXC1, FOXG1, FOXH1, FZD10, GLI2, GLI3, GPR56, GPSM2, HCCS, HESX1, HNRNPU, IGBP1, IGFBP1, ISPD, ITPA, KAL1, KAT6B, KATNB1, KIAA1279, KIF14, KIF1A, KIF1B, KIF21A, KIF2A, KIF5C, KIF7, L1CAM, LAMB1, LAMC3, LRP2, MCPH1, MED12, MID1, NDE1, NFIB, NPC1, NR2F1, NSD1, NTRK1, NTRK3, OCEL1, OPA1, OTX2, PAFAH1B1, PAX6, PEX1, PHF10, PIK3R2, POLR3A, POLR3B, POMT1, POMT2, PTCH1, PTPRS, PYCR1, RAB3GAP1, RARS2, RELN, RFX3, ROBO1, ROBO3, RPS6KA3, RTTN, SATB2, SEPSECS, SHH, SIX3, SLC12A6, SOX2, SPOCK1, SRPX2, TBCD, TBCE, TCF4, TDGF1, TEAD1, THBS2, TMEM5, TSC1, TSC2, TSEN15, TSEN2, TSEN34, TSEN54, TUBA1A, TUBA8, TUBB, TUBB2A, TUBB2B, TUBB3, TUBB4A, TUBG1, VAX1, VRK1, WDR47, WDR62, ZBTB18, ZEB2, ZIC2.

**Case 2**

Genomic DNA was extracted from venous peripheral blood lymphocytes, according to a standard phenol-chloroform procedure. The extracted DNA was amplified by PCR with oligonucleotides specific for exons 1-10 of the *ABCD1* gene (ATP-binding cassette, sub-family D, member 1) cr. Xq28. Exons and intron–exon boundaries were analyzed by direct sequence analysis and compared with the reference sequence (NM_000033.3 / NP_000024.2).

Subsequently, whole exome was performed on patient 2, using the SureSelect Human All Exon v7 (Agilent Technologies); the libraries of the patient were run on a NextSEQ500 according to the manufacturer's instructions (Illumina, San Diego, CA, USA).

ANNOVAR was used for annotation against the RefSeq and the Single Nucleotide Polymorphism databases. The filtering strategy we applied led us to select only variants that are located in the coding regions, including the splice site, that exhibited a minor allele frequency of less than 1% or that were not present in variant databases, including those of the 1000 Genomes Project, the Exome Aggregation Consortium (ExAC) and the NHLBI exome sequencing project ESP6500. Synonymous variants were excluded.

A recessive model was first used and *de novo* variants were also searched.

Variants were tested against different databases: 1000 genomes, Exome Variant Sever, dbSNP, HGMD (Human Gene Mutation Database) and ExAC databases. To confirm the pathogenicity of the mutations, we used web-based prediction programs: DANN (Bioinformatics 2015;31:761-763), Mutation taster (http://mutationtaster.org), SIFT (http://sift.jcvi.org), PROVEAN (http://provean.jcvi.org/), METASVM, FATHMM-MKL (http://fathmm.biocompute.org.uk.html) and PolyPhen-2 (http//genetics.bwh.harvard.edu/pph2) and Human Splicing Finder v.3.1 (http://www.umd.be/HSF3/).
